# The quiet transformation of higher education in the AI era

**DOI:** 10.12688/openreseurope.20715.1

**Published:** 2025-08-22

**Authors:** Nora Pireci Sejdiu, Sejdi Sejdiu

**Affiliations:** 1 Department of Computer Science, University of Prishtina, Prishtinë, Kosovo, 10000, Kosovo (Serbia and Montenegro); 2 Department of English Language and Literature, University of Prizren, Prizren, Kosovo, 20000, Kosovo (Serbia and Montenegro)

**Keywords:** Generative Artificial Intelligence (GenAI), higher education, digital pedagogy, algorithmic literacy, academic integrity, educational policy, ChatGP, AI ethics, digital inequality, assessment innovation

## Abstract

This paper explores the subtle yet profound transformations occurring in higher education as a result of advancements in artificial intelligence (AI). This paper critically examines the implications of GenAI for teaching, learning, assessment, and institutional policy. Through an interdisciplinary lens, the study explores how AI challenges traditional academic practices while offering opportunities for innovation. Key themes include the redefinition of digital literacy into algorithmic literacy, the ethical dilemmas facing educators and students, and the growing urgency to address digital inequality. The paper highlights the need for proactive institutional strategies that balance innovation with integrity, calling for inclusive policies, ethical frameworks, and the development of critical AI literacies. This article positions GenAI as both a disruptor and enabler, offering strategic guidance for how higher education institutions can navigate and evolve amid rapid technological change.

## Introduction

A quiet but profound transformation is taking place on campuses around the world. Imagine walking into a university classroom in 2025. It is not always visible in the lecture halls or the libraries, but the invisible presence of artificial intelligence in the daily academic lives of students. Generative AI tools like ChatGPT have become as ubiquitous as Wi-Fi, quietly shaping how students research, write, and even think. Yet, as these digital assistants become indispensable to students, many educators, especially those less familiar with the technology or less comfortable in English, find themselves on the other side of a growing digital divide.

This divide is not just about who uses AI, but how it is used, and what it means for the very heart of higher education. Are we still teaching students to think, or simply to prompt? Are assessments measuring understanding, or just the clever use of algorithms? These questions are no longer theoretical; they are urgent, practical, and deeply consequential.

This transformation is not evenly distributed. While students, digital natives in many respects, have swiftly adopted AI tools for everything from drafting essays to summarizing readings and even brainstorming research topics, many educators, particularly those less familiar with English or less comfortable with technology, find themselves on the other side of a widening digital divide ( Huang, 2023). In this new world, the old certainties of textbooks, lectures, and traditional assessments are being questioned. The university, once the undisputed center of knowledge and learning, must now reimagine its role in an era where information is everywhere and intelligence is, increasingly, artificial.

Surveys reveal the scale of this shift: by late 2023, more than half of college students in the United States reported using AI for assignments, with 86% of that use going undetected by instructors ( BestColleges, 2023; Intelligent.com, 2024). The line between authentic learning and algorithmic assistance has blurred, raising profound questions about the meaning of academic achievement. The experience of using AI is not uniform. Students from well-resourced backgrounds, with access to the latest devices and stable internet, can harness the full power of these tools. Others, perhaps struggling with connectivity or lacking digital literacy, may find themselves at a disadvantage. The promise of AI as an equalizer is real, but so is the risk that it will deepen existing inequalities ( Chiu *et al.*, 2021).

## The expanding universe of AI: beyond text generation

The power of generative AI extends far beyond the writing of essays. Machine translation tools like DeepL and Google Translate have made academic resources accessible across language barriers, opening new avenues for cross-cultural learning ( Deng & Yu, 2022).

Knowledge graphs help students and researchers visualize complex relationships, revealing patterns and connections that would otherwise remain hidden ( Ji *et al.*, 2022).

As these technologies mature, the potential for personalized and adaptive learning grows. Imagine a classroom where each student receives tailored feedback, or a laboratory where AI simulations allow for safe experimentation and exploration. AI can help educators design dynamic curricula, adapt to diverse learning styles, and offer support that would be impossible for a single human instructor ( Baidoo-Anu & Ansah, 2023; Michel-Villarreal *et al.*, 2023).

Yet the integration of these tools is not without risk. The digital divide between students and faculty threatens to widen, as not everyone has equal access or comfort with new technologies. The specter of algorithmic bias and the misuse of student data add further layers of complexity ( Igbokwe, 2023). The promise of AI is real, but so are the challenges.

## AI-driven personalized learning

As AI-driven personalized learning continues to evolve, it becomes imperative for higher education to not only focus on technological integration but also on the pedagogical shifts required to support diverse learning styles and needs. This transition calls for a reimagining of assessment methods, moving away from traditional standardized testing towards more dynamic, formative assessments that can adapt to individual student progress and provide real-time feedback. Such an approach aligns with the principles of inclusive education, fostering an environment where all learners can thrive, particularly those from underrepresented backgrounds ( Dumbuya, 2024). Moreover, the integration of AI tools in curriculum design can facilitate the development of critical thinking and problem-solving skills, essential in preparing students for the complexities of the modern workforce. By embracing these innovative assessment strategies, higher education institutions can enhance the learning experience and better prepare students for the challenges and opportunities of an AI-driven future.

This shift towards dynamic assessments not only enhances student engagement but also aligns with the need for equitable access to personalized learning experiences, ensuring all students benefit from AI advancements in education. This approach necessitates ongoing collaboration between educators and technologists to create adaptive learning environments that cater to diverse student needs while maintaining ethical standards in AI implementation.

## The educator’s dilemma: grading students or grading ChatGPT?

The arrival of generative AI has been both exhilarating and unsettling for educators. On the one hand, AI offers new possibilities for teaching: personalized feedback, automated grading, and the ability to create dynamic, adaptive learning experiences. On the other hand, it challenges some of the most fundamental assumptions about assessment and integrity.

The question that haunts many teachers is simple but profound: when I grade an assignment, am I assessing the student’s understanding, or the output of ChatGPT? The sophistication of AI-generated text is such that even experienced educators struggle to tell the difference ( Köbis & Mossink, 2021). Plagiarism detection tools, once a reliable safeguard, are increasingly ineffective against the nuanced prose of large language models ( Lim *et al.*, 2023; Zhai, 2023).

This uncertainty has prompted a range of responses. Some universities have embraced AI, publishing guidelines for its ethical and effective use ( Russell Group, 2024). Others have taken a more cautious stance, with bans and strict penalties for unauthorized use, as seen at Sciences Po in Paris and in the New York City Education Department ( Elsen-Rooney, 2023; France24, 2023). Yet even in institutions with clear policies, the day-to-day reality is often murky. When is AI use legitimate collaboration, and when is it academic dishonesty? The answer is rarely straightforward. This is not the first time that technology has upended the rituals of education. The introduction of calculators in mathematics classrooms sparked similar debates about fairness, skill, and the nature of learning. Over time, calculators were accepted, but only after educators reimagined what it meant to “show your work” and demonstrate understanding.

The story of AI in higher education echoes this earlier transition, but with important differences. Calculators affected a specific domain; AI touches everything. Where calculators automated arithmetic, AI can generate essays, solve problems, and even simulate conversations. The challenge is not just to adapt assessment, but to rethink the entire educational process.

## Rethinking assessment: What does it mean to learn?

Perhaps nowhere is the impact of AI felt more acutely than in the realm of assessment. Traditional methods, essays, take-home exams, and even some forms of online testing, are increasingly vulnerable to AI assistance. As a result, educators are rethinking what it means to measure learning. The focus is shifting from product to process, from rote memorization to critical thinking, creativity, and the ability to synthesize and apply knowledge.

This transformation is not without its challenges. Designing assessments that are both rigorous and resistant to AI manipulation requires creativity and a willingness to experiment. Some faculty are turning to oral defenses, collaborative projects, and reflective journals as ways to capture the nuances of student learning. Others are leveraging AI itself, using it to provide timely feedback and support, while remaining vigilant against its misuse.

The ultimate goal is to ensure that assessment remains fair, meaningful, and reflective of each student’s true capabilities. This means not only updating policies and practices, but also fostering a culture of integrity and trust-a culture in which the use of AI is transparent, ethical, and aligned with the values of higher education.

As AI becomes more entrenched in the fabric of higher education, new questions arise about sustainability. The environmental impact of training large AI models is significant, with substantial energy consumption and a growing carbon footprint ( Strubell *et al.*, 2019). Universities are beginning to reckon with these costs, seeking out energy-efficient solutions and encouraging responsible use of digital resources.

Equity, too, is a pressing concern. Not all students have equal access to AI tools or the skills to use them effectively. Without careful attention, the benefits of AI could accrue disproportionately to those who are already privileged, widening the gap between the haves and have-nots ( Chiu *et al.*, 2021). Addressing these challenges requires a commitment to inclusivity, ensuring that all members of the academic community have the support and resources they need to thrive in the AI era.

## New skill requirements for students

As universities adapt their curricula to incorporate AI tools, there is a pressing need to redefine the competencies that students must acquire to succeed in a rapidly changing job market. This includes not only technical skills related to AI and data analysis but also soft skills such as creativity, emotional intelligence, and adaptability, which are increasingly valued in the workplace. Furthermore, the ability to critically assess AI-generated content and engage in ethical discussions surrounding technology use becomes paramount, as students must navigate a landscape rife with algorithmic bias and misinformation. By fostering a curriculum that balances technical proficiency with these essential soft skills, higher education can better prepare students to thrive in an environment where AI is ubiquitous, thereby ensuring that they are not only consumers of technology but also ethical stewards of its application in society ( Asrifan *et al.*, 2024; Bibi, 2024). This holistic approach to skill development will ultimately contribute to a more equitable and innovative workforce, capable of leveraging AI for positive social impact.

## Institutional responses: A patchwork of policy

The integration of AI into higher education has prompted significant policy shifts, as institutions strive to balance innovation with ethical considerations and academic integrity. Some universities, like California State University, have embraced a proactive approach, partnering with AI developers to roll out customized tools across their campuses. These initiatives aim to provide personalized tutoring, streamline administrative tasks, and support research, all while maintaining robust oversight to ensure ethical use ( Reuters, 2025).

Elsewhere, caution prevails. Concerns about cheating and the erosion of academic standards have led some universities to restrict or even ban the use of AI tools in coursework. These divergent responses reflect the complexity of the issues at stake, as well as the absence of a clear consensus on best practices ( Buolamwini, 2024).

What is emerging, however, is a recognition that policy cannot be static. The landscape is evolving too quickly, and what works today may be inadequate tomorrow. Universities are beginning to see the value in continuous dialogue, sharing best practices, and adapting their guidelines as technology and social norms shift ( Sullivan *et al.*, 2023).

## The ethical tightrope: innovation and responsibility

The rapid evolution of AI has outpaced the development of regulatory and policy frameworks. Universities find themselves in a “grey area,” where the rules are unclear and the stakes are high ( Fitria, 2021). On one hand, there is an imperative to harness the power of AI to enhance learning and research. On the other, there is a responsibility to safeguard academic integrity, protect student privacy, and ensure that no one is left behind.

The role of the educator is changing. No longer simply transmitters of knowledge, faculty are increasingly called upon to act as facilitators, helping students navigate a world where information is abundant and easily accessible ( Gentile *et al.*, 2023). This shift demands new pedagogical strategies and a commitment to ongoing professional development ( Kiryakova & Angelova, 2023). It also requires a willingness to engage with the ethical dilemmas that AI presents, from questions of authorship to the potential for deepening educational inequalities.

Universities are beginning to grapple with these challenges, experimenting with new forms of assessment that go beyond the traditional essay. Oral examinations, project-based learning, and in-class presentations are gaining traction as ways to ensure that students are demonstrating genuine understanding, rather than simply passing off AI-generated work as their own ( Ateeq *et al.*, 2024). At the same time, some institutions are exploring the use of AI itself as a tool for assessment, providing formative feedback and helping to detect instances of plagiarism, always with human oversight ( AlAfnan *et al.*, 2023).

As part of the evolving educational landscape, developing algorithmic literacy among both students and educators is no longer optional - it is essential. Algorithmic literacy refers to the ability to understand, critique, and responsibly engage with algorithm-driven systems, such as generative AI tools. This includes recognizing how algorithms operate, what data they are trained on, how outputs are generated, and how these systems may encode bias or reinforce systemic inequalities ( Gran *et al.*, 2021; Long & Magerko, 2020).

Without this knowledge, users may perceive AI-generated responses as objective or authoritative, failing to recognize the embedded assumptions, training limitations, or ethical blind spots that underlie these technologies. For example, large language models like ChatGPT are trained on vast and diverse data sets, but these corpora often reflect dominant cultural, linguistic, and ideological biases ( Binns, 2018; Gebru *et al.*, 2021). This can influence not only the content students receive but also the framing of knowledge itself.

Teaching algorithmic literacy empowers learners to interrogate these outputs critically, fostering more reflective engagement with digital tools. It also equips educators to guide students in distinguishing between AI-generated content and original academic work, reinforcing the values of critical inquiry and intellectual autonomy.

Some scholars argue that algorithmic literacy should be treated as a core academic skill, on par with digital literacy and information literacy, within university curricula ( D'Ignazio & Klein, 2021). Embedding this literacy into academic programs can help demystify AI tools, promote ethical awareness, and reduce the risk of misuse or overreliance. As AI continues to shape educational experiences in subtle but profound ways, algorithmic literacy provides the necessary scaffolding to ensure that both educators and students remain informed, empowered, and critically engaged participants in the learning process.

## The road ahead: writing the next chapter

The story of AI in higher education is still being written. Each institution, each classroom, and each student is part of an ongoing experiment, testing the boundaries of what is possible and what is desirable. The choices made today will shape the university of tomorrow, influencing not only how knowledge is created and shared, but also what it means to be educated in a world where machines can think, write, and even create.

The path forward will not be easy. It will require courage, creativity, and a willingness to engage with complexity. But it will also offer opportunities for renewal and reinvention, as universities rediscover their purpose and reaffirm their commitment to the values that have always defined higher education: curiosity, integrity, and the pursuit of truth.

In this unfolding narrative, the challenge is not simply to keep pace with technology, but to shape it-to ensure that the tools we create serve the broader goals of learning, equity, and human flourishing. The AI revolution in higher education is not just a story of machines, but a story of people: students, educators, and communities, working together to imagine a future in which technology enhances, rather than diminishes, the human spirit.

## Conclusion

As we look ahead to the next decade, the integration of AI in higher education will likely necessitate a re-evaluation of institutional accreditation processes, ensuring they align with the evolving landscape of technology-enhanced learning. Accreditation bodies may need to establish new criteria that not only assess traditional academic rigor but also evaluate how effectively institutions are incorporating AI into their curricula and fostering ethical practices among students and educators alike. This shift could promote a more holistic view of educational quality, encouraging institutions to prioritize innovative teaching methods and ethical considerations in technology use. Furthermore, as the demand for AI literacy grows across various sectors, institutions that proactively adapt their accreditation standards will likely attract a broader student base, positioning themselves as leaders in the educational field and driving a more equitable workforce prepared for the challenges of an AI-driven future ( Ibanga, 2024).

## Ethics and consent

Ethical approval and consent were not required.

## Data Availability

No data are associated with this article.
