# Supplementary material for: The quiet transformation of higher education in the AI era
Source: Open Res Eur. 2025 Aug 22;5:249. [Version 1] doi: 10.12688/openreseurope.20715.1 (PMC12438950; doi:10.12688/openreseurope.20715.1)
Supplement: Peer review history

# Reviewer report: Reviewer response for version 1

**DOI:** 10.21956/openreseurope.22406.r59025

**Published:** 2025-09-15

**Authors:** Nuhi Bllaca

**Affiliations:** 1 Department of English and American Studies, University of Vienna, Vienna, Austria

In this essay, the authors make a valuable contribution by exploring the role of Generative AI (GenAI) in higher education. Their discussion addresses a key gap in the literature on AI tools’ influence in this field, highlighting both the opportunities and challenges of tools such as ChatGPT, particularly their impact on teaching, learning, and assessment. The essay underscores the responsibility of universities to ensure equitable access to AI technologies, establish clear policies that promote constructive use, and provide training programs that develop students’ AI literacy. Nonetheless, there are a few limitations worth noting: (a) It does not situate its analysis within a specific country or institutional context, which reduces the applicability of its insights; and (b) its reliance on secondary sources makes its claims less robust, underscoring the need for future empirical studies to validate these arguments

Is the argument persuasive and supported by appropriate evidence?

Yes

Is the work clearly and cogently presented?

Yes

Is the topic of the essay discussed accurately in the context of the current literature?

Yes

Does the essay contribute to the cultural, historical, social understanding of the field?

Yes

Reviewer Expertise:

Peer interaction, Collaborative writing, Computer-assisted language learning (CALL), Written corrective feedback, and the role of GenAI tools (ChatGPT) in L2 writing.

I confirm that I have read this submission and believe that I have an appropriate level of expertise to confirm that it is of an acceptable scientific standard.

# Reviewer report: Reviewer response for version 1

**DOI:** 10.21956/openreseurope.22406.r59022

**Published:** 2025-09-12

**Authors:** Mirela Alhasani

**Affiliations:** 1 Department of Applied Linguistics, Epoka University, Tirana, Albania; 2 Center for European Studies, Epoka University, Tirana, Albania

This article tackles a highly sensitive and important research puzzle about the role of AI in higher education. Most significantly, the authors elaborate in a smooth and clear balance not only the advantages of AI in education but also the dilemmas for the academic community in its proper usage for the quality teaching and learning process. Authors have succeeded in justifying the gap and the urgent need for the development of critical AI literacies and inclusive policies by higher education policies in a rapid time amid rapid technological changes.  

 However, the paper needs to mention its limitations in a discussion at a general macro level without a specific reference to a country or region case study. it needs to be mentioned in the recommendation concluding points that the analysis remains at an overall theoretical level, paving the way to country-to-country investigation and policy analysis for AI usage.

Is the argument persuasive and supported by appropriate evidence?

Yes

Is the work clearly and cogently presented?

Yes

Is the topic of the essay discussed accurately in the context of the current literature?

Yes

Does the essay contribute to the cultural, historical, social understanding of the field?

Yes

Reviewer Expertise:

Higher education pedagogy

I confirm that I have read this submission and believe that I have an appropriate level of expertise to confirm that it is of an acceptable scientific standard.

## References

[1] : The quiet transformation of higher education in the AI era. *Open Research Europe* .2025;5: 10.12688/openreseurope.20715.1 10.12688/openreseurope.20715.1 40386579
